# Transcription factor binding to *Caenorhabditis elegans* first introns reveals lack of redundancy with gene promoters

**DOI:** 10.1093/nar/gkt858

**Published:** 2013-09-24

**Authors:** Juan I. Fuxman Bass, Alex M. Tamburino, Akihiro Mori, Nathan Beittel, Matthew T. Weirauch, John S. Reece-Hoyes, Albertha J. M. Walhout

**Affiliations:** ^1^Program in Systems Biology, Program in Molecular Medicine, University of Massachusetts Medical School, Worcester, MA 01605, USA, ^2^Center for Autoimmune Genomics and Etiology (CAGE), Cincinnati Children’s Hospital Medical Center, Cincinnati, OH 45229, USA and ^3^Divisions of Rheumatology and Biomedical Informatics, Cincinnati Children's Hospital Medical Center, Cincinnati, OH 45229, USA

## Abstract

Gene expression is controlled through the binding of transcription factors (TFs) to regulatory genomic regions. First introns are longer than other introns in multiple eukaryotic species and are under selective constraint. Here we explore the importance of first introns in TF binding in the nematode *Caenorhabditis elegans* by combining computational predictions and experimentally derived TF–DNA interaction data. We found that first introns of *C. elegans* genes, particularly those for families enriched in long first introns, are more conserved in length, have more conserved predicted TF interactions and are bound by more TFs than other introns. We detected a significant positive correlation between first intron size and the number of TF interactions obtained from chromatin immunoprecipitation assays or determined by yeast one-hybrid assays. TFs that bind first introns are largely different from those binding promoters, suggesting that the different interactions are complementary rather than redundant. By combining first intron and promoter interactions, we found that genes that share a large fraction of TF interactions are more likely to be co-expressed than when only TF interactions with promoters are considered. Altogether, our data suggest that *C. elegans* gene regulation may be additive through the combined effects of multiple regulatory regions.

## INTRODUCTION

The precise expression of genes in space and time plays a central role in development, homeostasis and response to environmental cues. Gene expression is regulated through the binding of transcription factors (TFs) to regulatory genomic regions such as promoters and enhancers. The regulation of transcription is relatively simple in single cell eukaryotes such as the yeast *Saccharomyces cerevisiae,* where TFs bind promoters immediately upstream of the transcription start site (TSS) (1). However, in more complex multicellular organisms such as the fruit fly *Drosophila melanogaster* and mammals, additional genomic sequences known as enhancers can affect gene expression. Such enhancers can be located at distal sites, far from the TSS, and are often found in introns (2,3). Enhancers are rapidly being identified, for instance, using specific combinations of histone marks or binding of the p300 cofactor (4,5). However, little is known about the repertoire of TFs that interact with enhancers or the relative contribution of enhancers to the expression of individual genes.

The nematode *Caenorhabditis elegans* is a primary model system for the study of development, as well as physiological processes such as fat storage, dietary response and aging (6–8). The *C. elegans* genome encodes 937 predicted TFs (9). Although numerous classical enhancers have been identified in the fly, little is known about the complexity of gene regulation in *C. elegans*.

Interactions between regulatory DNA sequences and TFs can be experimentally identified using either TF-centered (protein-to-DNA) or gene-centered approaches (DNA-to-protein) (10,11). TF-centered methods such as chromatin immunoprecipitation (ChIP) have been used to identify *in vivo* binding events for a relatively small number of *C. elegans* TFs and, usually, only in a single developmental stage (12). These studies have found that TFs generally bind sequences located within a few hundred nucleotides from the TSS. Although there is a clear bias for regions upstream of TSSs (12,13), there is also evidence of significant TF binding in downstream regions, in particular for some TFs, including UNC-130 and CEH-14 (12). Furthermore, conserved TF binding sites (TFBSs) that are required for appropriate gene expression have been identified within the introns of *C. elegans* genes, in particular the first intron (14–17). However, these observations were based on a few examples, and the abundance of TFBSs in the first intron of *C. elegans* genes remains to be determined.

In contrast to ChIP, gene-centered yeast one-hybrid (Y1H) assays can be used to identify the repertoire of TFs that can bind to a DNA sequence of interest (18,19). Collectively, Y1H assays have thus far identified binding events for ∼35% of all predicted *C. elegans* TFs (20–24). Y1H assays use a standardized format that detects interactions in the milieu of the yeast nucleus. Thus, even though these assays do not directly identify *in vivo* interactions, they are much less condition-dependent, enabling the detection of interactions that occur in different stages in the animal’s lifetime or in only a few cells.

It is thought that the first intron plays a substantial role in the regulation of gene expression in *C. elegans*. However, with the exception of 43 TFs that have been profiled genome-wide by ChIP (5% of all TFs), the propensity of first introns to interact with TFs has been largely unexplored. Here, we use a combined experimental and computational approach to investigate TF binding to *C. elegans* (first) introns. We find that the first intron is generally longer than other introns, which is in agreement with previous observations (25). We further find that first introns are particularly long for certain types of genes, including G–protein-coupled receptors (GPCR), endoglin/CD105 genes and homeodomain TFs. The length of the first intron is generally more conserved between *C. elegans* and *Caenorhabditis briggsae* than the length of other introns, which indicates that a long first intron length is under selective pressure. The first intron generally binds more TFs in ChIP assays and harbors more predicted TF interactions that are conserved between *C. elegans* and C. *briggsae* than other introns. We use Y1H assays to systematically identify the TFs that can bind first introns of varying length and compare the data obtained with the TFs that can bind the cognate gene promoters. Perhaps not surprisingly, we find a correlation between first intron length and number of TF interactions. Remarkably, however, TFs that bind the first intron are usually distinct from those binding the promoter of the same gene. Finally, we show that shared interactions between genes are more predictive of co-expression when both the promoter and the first intron are considered. Altogether, these observations suggest that gene regulation may be additive through multiple regulatory regions.

## MATERIALS AND METHODS

### ‘Conventional gene’ set

Genomic sequences and annotations were downloaded from ftp.wormbase.org on 9 December 2011. Annotations were amended to include the DNA sequence for each fragment as well as the exon or intron number. Fragments were numbered 5′–3′ based on their genomic location. Next, genes were individually analyzed for the presence of introns in the 5′-UTR (i.e. one or more introns upstream of start codon), alternative start sites (i.e. two or more distinct first coding exons) and presence downstream in an operon (i.e. located in an operon, not the first gene). Genes that were negative for all three criteria were defined as ‘conventional genes’ (15 621 genes). Promoter regions were defined from the start codon to up to 2-kb upstream unless there was a neighboring gene closer, in which case the region was extended until the start codon or the 3′-end of the neighboring gene.

### Length conservation analysis

Gene orthology was based on InParanoid data (26). Individual coding exons for each *C. elegans* conventional gene were compared to all coding exons of the *C. briggsae* ortholog to determine the homologous segment using tBlastx. Matches were tabulated and genes where contiguous one-to-one alignments could be accurately detected were used for downstream analyses. The analysis of length conservation was performed for genes that have an identical exon–intron structure (i.e. all exons are homologous between the two species and have the same order) in *C. elegans* and *C*. *briggsae* (3404 genes) to be able to determine and compare length conservation for all introns. For length conservation analysis, only genes that have a first intron longer than 500 bp in at least one of the species were considered (942 genes).

### ChIP binding site analysis

ChIP interaction data from the modENCODE Project (12) were downloaded from http://intermine.modencode.org/ on 29 April 2012. The midpoint of the ChIPped region was defined as the binding site. Gene fragments were analyzed individually to determine whether the TF ChIP peak midpoints were located between the start and end of the fragment.

### Predicted binding site analysis

Predicted TFBSs were determined using the energy scoring system used by Binding Energy Estimates using Maximum Likelihood (BEEML) (27) to score 33 position weight matrices (PWMs) for 29 *C. elegans* TFs that belong to a broad range of TF families. We used a threshold of 0.09 as previously determined (24). A predicted TF interaction was regarded as conserved in a DNA gene region if a TF was predicted to interact with both the *C. elegans* and the *C. briggsae* DNA fragments. The number of conserved predicted TF interactions was calculated as the number of TFs predicted to interact with the *C. elegans* and *C. briggsae* orthologous regions for genes with identical exon–intron structure. DNA fragments that do not have any predicted site in either species were excluded from the analysis.

### Gene ontology analysis

*Caenorhabditis elegans* conventional genes with first introns longer than 250 bp were analyzed for gene family enrichment (gene families defined according to InterPro) using DAVID Bioinformatics Functional Annotation tool (28). The gene families overrepresented in the set of genes with long first introns were then analyzed for the length of their first and non-first introns, conserved intron length, conserved predicted TF interactions and TF interactions obtained from ChIP experiments.

### eY1H assays

We selected the first introns of 227 *C. elegans* conventional genes to use as baits in eY1H assays. Genes were binned according to their first intron length (<65, 65–128, 129–256, 257–512 and >512) and 40–50 genes were randomly selected from each bin. The first introns of the selected genes were cloned as previously described for promoter fragments (29). Briefly, first introns were amplified from *C. elegans* (N2) genomic DNA by polymerase chain reaction, cloned into a gateway entry vector and subsequently transferred into the *HIS3* and *LacZ* Y1H reporter Destination vectors. The reporter constructs were integrated into the genome of *S. cerevisiae* (Y1H-aS2) to create Y1H bait strains as described (29). In total, 164 first intron Y1H bait strains were successfully generated. Primer sequences and detailed information about Y1H bait strains are provided in Supplementary Table S1. Yeast bait strains corresponding to gene promoters were generated using the promoterome resource (30). eY1H assays were performed as described (9). Plates were analyzed automatically using the SpotOn tool (9), and data were manually curated (Supplementary Table S2). Interactions that were detected in two of four replicates were considered positive. Auto-active and low-confidence DNA baits (i.e. baits with non-uniform background reporter expression) (10 for first intron baits) were removed from further analysis.

### TF binding and co-expression for homeodomain genes

Yeast DNA strains were generated for the first introns of 24 homeodomain genes and screened using the eY1H platform (Supplementary Table S1). Promoter interactions for the same homeodomain genes were obtained from Reece-Hoyes (24). The protein–DNA interaction (PDI) similarity between two genes was determined by considering only promoters, only first introns or both, using the Jaccard index formula:

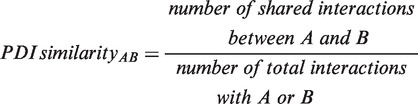



The co-expression correlations between the homeodomain genes were obtained from 123 expression-profiling experiments available in the worm Serial Pattern of Expression Levels Locator (SPELL) database (31). For each pair of genes, this co-expression score represents the similarity in expression levels across expression-profiling experiments.

### In-degree distribution

The promoter and first intron in-degree (number of TF interactions per regulatory element) distributions were modeled using the exponential distribution as previously shown for promoters in several species (20,21,32,33):

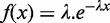

where *f* is the probability density distribution, *x* is the number of interactions and 1/*λ* is the average number of interactions.

The promoter + first intron in-degree distribution was modeled with a hypo-exponential distribution (34). This distribution is obtained when summing two or more variables randomly sampled from independent exponential distributions:

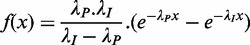



The λ values were estimated for the promoter (λ_P_ = 0.145) and the first intron (λ_I_ = 0.3) in-degree distributions and used to determine the expected hypo-exponential distribution for the promoter + first intron in-degree.

## RESULTS AND DISCUSSION

### First intron length conservation

**Figure 1.**
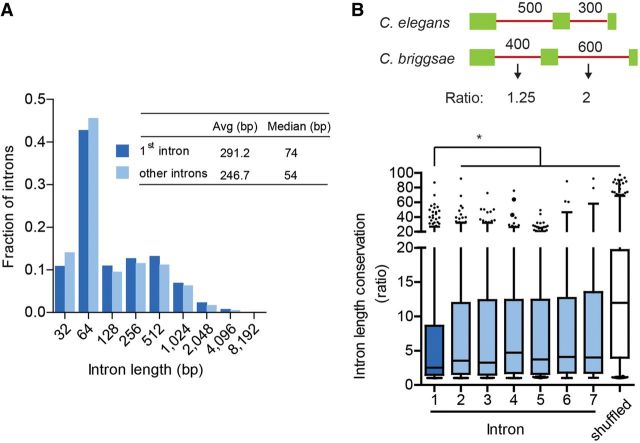
Intron length and length conservation. (**A**) Intron length distribution for *C. elegans* conventional genes. (**B**) Ratio between *C. elegans* and *C. briggsae* intron lengths (larger over smaller) determined between orthologous introns of orthologous genes. The white box corresponds to the ratio between the lengths of random pairs of non-orthologous first introns. *P-value < 0.01, Mann–Whitney *U* test.

Previous reports indicate that first introns are longer than other introns and are under more selective constraint in a variety of organisms (25,35). We re-analyzed intron length in *C. elegans* excluding genes that have an alternative TSS downstream from the first intron, genes whose first intron is located within the 5′ untranslated region and genes located downstream in operons (because their first introns are not the intron closest to the TSS). We named the remaining set of genes ‘conventional genes’ (76% of *C. elegans* protein-coding genes). As shown in Figure 1A, first introns of *C. elegans* conventional genes are longer than other introns (median length of 74 versus 57 bp, *P* < 0.0001), confirming previous observations (25).

If first introns are important for gene regulation, one would expect that the length of long first introns is more conserved than the length of long non-first introns, as they could potentially contain more TFBSs and would thus be more sensitive to deletions. To test this prediction, we compared the intron length ratio between conventional genes of two related nematode species, *C. elegans* and *C. briggsae,* using genes that have the same exon–intron structure in both species (3404 genes) and whose first intron is ≥500-bp long in at least one species (942 genes). Genes with different exon–intron structure were not included in the analysis to be able to compare intron length conservation between introns at different positions. Additionally, introns that are shorter than 500 bp in both species were excluded from the analysis, as length conservation in this case may only reflect a general tendency for worm introns to be short. Briefly, we divided the length of the longer of the two introns by that of the shorter one. When intron length is conserved, the intron length ratio is close to 1, whereas larger discrepancies in intron length result in values >1 (Figure 1B). By using this metric, we found that the length of first introns is significantly more conserved than the length of other introns (*P* < 0.01). To confirm that this observation is not specific to the *C. elegans* and *C. briggsae* comparison, we compared 100 randomly selected *C. elegans* genes with orthologs in *C. remanei* and observed a 28% increase in conservation of first intron length relative to the second intron (data not shown). These results suggest that there is evolutionary pressure to maintain the length of first introns, which points to a functionally important role such as the regulation of gene expression.

### TF binding to first introns

**Figure 2.**
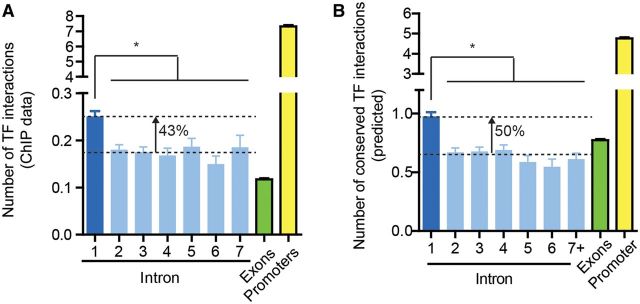
TF binding to introns. (**A**) PDIs detected in ChIP assays from the modENCODE Project for conventional genes were partitioned into different gene regions. The average number (±SEM) of TFs that interact with each region is plotted. (**B**) Average number (±SEM) of predicted TF interactions that are conserved between *C. elegans* and *C. briggsae* orthologous gene regions. *P-value < 0.001, Mann–Whitney *U* test.

To test the potential importance of the first intron in gene regulation, we used TF binding as a proxy. We first evaluated the frequency of TF interactions with different gene regions using ChIP data for 43 TFs obtained from the modENCODE Project (12). We found that, on average, first introns of conventional genes bind 43% more TFs than other introns (Figure 2A). This increased TF binding cannot solely be explained by length difference because first introns of conventional genes are on average only 18% longer than other introns (Figure 1A). Further, it is most likely that the number, rather than the density, of TF interactions is more functionally relevant. For instance, a 1-kb intron with 10 TF interactions will probably have a greater impact on gene regulation than a 50-bp intron with only one TF interaction.

Regulatory regions are often more conserved between related species than other non-coding regions (36). In fact, sequence conservation has proven useful to identify enhancers in flies and vertebrates (37,38), and conserved TFBSs are more likely to have functional relevance as evidenced by ChIP assays (37). Additionally, DNA binding motifs for TFs are generally conserved between species (39). Therefore, we hypothesized that first introns might harbor more conserved (predicted) TF interactions than other introns. Predicted TFBSs were determined using an energy scoring system similar to that used by BEEML (27) with 33 PWMs for 29 *C. elegans* TFs (24). By comparing predicted TFBSs between *C. elegans* and *C. briggsae*, we found that first introns harbor 50% more TF interactions that are predicted to be present in both species than other introns (Figure 2B). Taken together, the observations that first introns are longer, that their length tends to be more conserved and that they harbor more experimentally defined as well as computationally predicted conserved TF binding events, indicate that first introns are more important contributors to gene regulation than other introns.

### Redundancy between promoters and first introns

**Figure 3.**
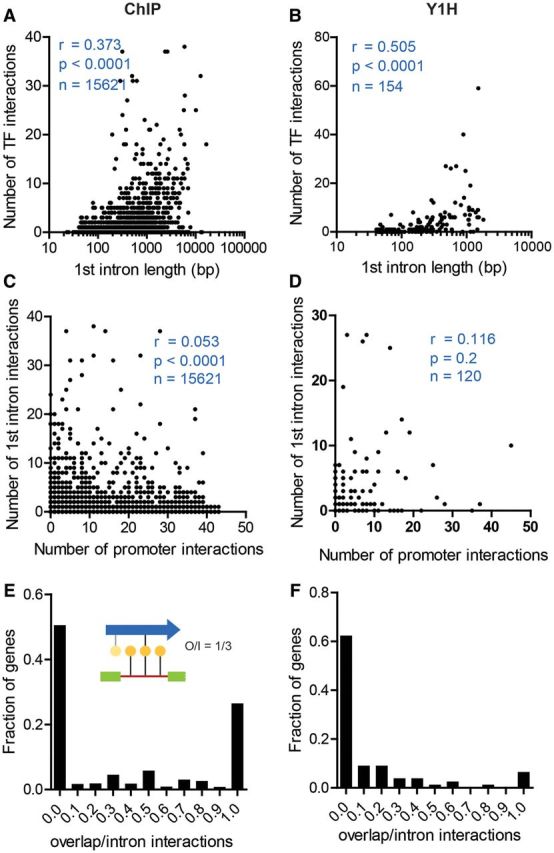
Redundancy between promoter and first intron TF interactions. The number of TFs that interact with the first introns of *C. elegans* genes in (**A**) ChIP or (**B**) eY1H assays is plotted against the first intron size. Correlation between the number of promoter interactions and the number of first intron interactions determined in (**C**) ChIP or (**D**) eY1H assays. For each gene, the number of shared TF interactions determined by (**E**) ChIP or (**F**) eY1H between first introns and promoters was divided by the number of intron interactions to calculate the overlap/intron (O/I) interactions.

First introns bind on average only 3.4% of the number of TFs compared with promoters as determined by ChIP assays (Figure 2A). However, given the broad distribution in intron length, TF binding may be more relevant for long first introns that potentially harbor a larger number of TFBSs. There is a significant positive correlation between the number of TF interactions detected in ChIP assays and the length of the first intron (Figure 3A, r = 0.373, *P* < 0.0001), with first introns longer than 500 bp having on average 30-fold more TF interactions than first introns shorter than 100 bp.

Genome-wide ChIP assays have been performed with only 5% of all *C. elegans* TFs and, in most cases, only in a single developmental stage and laboratory condition (12,13). To be able to characterize TF interactions with first introns in a condition-independent and TF-wide manner, we used enhanced Y1H (eY1H) assays. This technology uses a 1536-colony platform to interrogate interactions with 834 *C. elegans* TFs (89% of all 937) in a high-throughput manner (9). We successfully screened 154 first introns of a broad range of lengths (41–1812 bp). Of 133 210 intron-TF combinations tested, 585 scored positively (0.4%), involving 98 first introns (56 first introns had no interactions) and 166 proteins. These include 161 TFs and five unconventional DNA binding proteins, proteins that lack a recognizable DNA binding domain but that have previously been detected in Y1H assays (20,21). Consistent with the results obtained from ChIP assays, there is a significant positive correlation between the length of the first intron and the number of TFs bound (r = 0.505, *P* < 0.0001, *n* = 154, Figure 3B). Most first introns shorter than 100 bp bind no or very few TFs, whereas most long first introns bind multiple TFs. Projecting these values to all *C. elegans* conventional genes, 57% of the genes are expected to have at least one TF interacting with their first intron, and the average number of interactions per first intron is ∼2.9 (average length = 291.2 bp). Although this number is lower than the average number of TFs that bind gene promoters (average length = 1340 bp, average interactions = 8.8) (24), our results suggest that a large number of genes may bind TFs within the first intron.

Given that some first introns, in particular longer ones, bind a large number of TFs, we hypothesized that these introns may compensate for low TF binding to the cognate promoter. To test this hypothesis, we compared the number of ChIP interactions between promoters and first introns on a gene-by-gene basis (Figure 3C). We did not detect a negative correlation between the number of first intron and promoter interactions, suggesting that TF binding to both regions is independent. To directly compare the two regions in a gene-centered manner, we performed eY1H assays with the promoters of the genes whose first introns were interrogated in eY1H assays. We successfully screened 120 promoters and detected 827 interactions involving 167 proteins. We did not observe a significant correlation between the number of TFs that bind the first intron and the number of TFs that bind the promoter of each gene tested, further supporting the results obtained by analyzing ChIP data (Figure 3D). Altogether, these observations suggest that first introns do not or only rarely compensate for a low number of promoter interactions, but are instead independent.

We reasoned that TF interactions with first introns could contribute to gene regulation in two ways. First, TFs that bind the first intron could be overlapping with those that bind promoters, resulting in redundancy and, as a consequence, a robust regulation of gene expression (40,41). Second, promoters and first introns could regulate gene expression in an independent manner by binding different TFs. This could result in additive or cooperative regulation within the same cell or contribute independently to gene expression in different tissues/cells. To distinguish between these two alternatives, we compared TF interactions with first introns and promoters on a per gene basis by calculating the fraction of first intron interactions that were shared with their respective promoter (Figure 3E). In ChIP experiments, most genes show no overlap between first intron and promoter interactions suggesting that the regulatory role of first introns is, in general, not redundant with that of the promoters (Figure 3E). However, for 27% of genes that have TFs binding the first intron, all TFs bound also interact with the respective promoter. It is not possible to discriminate between direct and indirect interactions in ChIP assays (42). For instance, TF binding events that occur only at the promoter may also be detected with the first intron when the two regions interact via DNA looping (43). Interactions detected by eY1H assays are more likely direct. We found that the fraction of genes with complete overlap in TF binding to their first intron and their promoter as detected by eY1H assays is much lower (Figure 3F). Altogether, these observations suggest that TFs that bind first introns are largely distinct from those binding the promoter.

### Combined promoter and first intron TF interactions predict co-expression better than either alone

**Figure 4.**
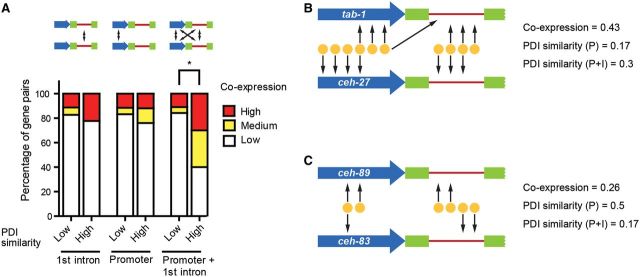
Regulatory consequences of TF–first intron interactions. (**A**) TF interactions with the promoters and first introns of 24 homeodomain genes were determined by eY1H. For each pair of genes, the PDI similarity was determined using the Jaccard index and partitioned into low (J < 0.3) or high (J ≥ 0.3). Gene-pairs were partitioned according to their co-expression correlation into low (co-expression < 0.2), medium (0.2 ≤ co-expression < 0.5) and high (0.5 ≤ co-expression). *P < 0.01, Fisher’s exact test. (**B**) Example of two homeodomain genes, *tab-1* and *ceh-27*, that have a higher PDI similarity when including first intron interactions. (**C**) Example of two genes, *ceh-89* and *ceh-83*, that have a lower PDI similarity when including first intron interactions.

Gene regulation is achieved by the combinatorial action of TFs. We hypothesized that if two genes share a large fraction of TFs binding to their regulatory regions, they are more likely to be co-expressed than two genes that have different interacting TFs. To test this hypothesis, we focused on 24 homeodomain genes, a gene family enriched in long first introns (Table 1, see later in the text), for which we screened both the promoter (24) and the first intron in eY1H assays. For each pair of genes we calculated a PDI similarity score using the Jaccard index (J) by measuring the fraction of shared TFs relative to the total number of TFs for only first introns, only promoters or both. This index considers all the differential interactions between two genes, as they are all equally likely to contribute to a differential gene expression (44). We first partitioned the gene-pairs according to PDI similarity (low J < 0.3 or high J ≥ 0.3). Second, we partitioned the gene-pairs according to their co-expression correlations obtained from 123 expression-profiling experiments available in the worm SPELL database (31) in low (co-expression < 0.2), medium (0.2 ≤ co-expression < 0.5) and high (co-expression ≥ 0.5). We expected that gene-pairs with high PDI similarity would be enriched for high co-expression pairs relative to those with a low PDI similarity. When only promoter interactions were considered, there was no significant difference in co-expression between low and high PDI similarity gene-pairs (Figure 4A, *P* = 0.286). The same was true when only first intron interactions were considered (Figure 4A, *P* = 0.466). However, when promoter and first intron interactions were combined, gene-pairs with a high PDI similarity were enriched for gene-pairs with medium and high co-expression (Figure 4A, *P* = 0.0016). This observation provides further support for the notion that promoter and first intron interactions are not redundant, but rather may independently contribute to gene expression.

**Table 1.** *C. elegans* first intron length distribution

Term	Number of genes	First intron length		Other intron length		Benjamini *P*-value
		Average	Median	Average	Median	
Conventional genes^a^	15 621	291.2	74	246.7	57	
IPR000276:7TM GPCR, rhodopsin-like	143	582.2	256	252	78	3.22E-05
IPR006029:Neurotransmitter-gated ion-channel transmembrane region	63	412.6	192.5	304.3	91	0.0014
IPR001356:Homeodomain	70	442.9	263	495	148	0.0018
IPR001507:Endoglin/CD105 antigen	36	745.1	466.5	243.4	72	0.025
IPR000609:7TM GPCR, serpentine receptor class g (Srg)	60	364.7	293.5	166.8	54	0.026
IPR006209:EGF	23	550	411	236.6	59	0.035
IPR019425:7TM GPCR chemoreceptor, Srt, serpentine type	59	387.4	254	228	57	0.042

^a^Genes that have a unique TSS, that have their first intron located downstream of the translation start codon and that are either not located in an operon or they correspond to the first gene in the operon.

Only considering promoter interactions can result in either an overestimation or an underestimation of shared regulators. For example, *tab-1* and *ceh-27* have a relatively high co-expression correlation (0.43), although they have a low PDI similarity (0.17) if only promoter interactions are considered (Figure 4B). However, if first intron interactions are also included, the PDI similarity score increases to 0.3. Conversely, *ceh-83* and *ceh-89* have a relatively low co-expression correlation (0.26), although they have a high PDI similarity (0.5) when considering only promoter interactions, but have a low PDI similarity (0.17) if first intron interactions are included (Figure 4C).

### Network topology

**Figure 5.**
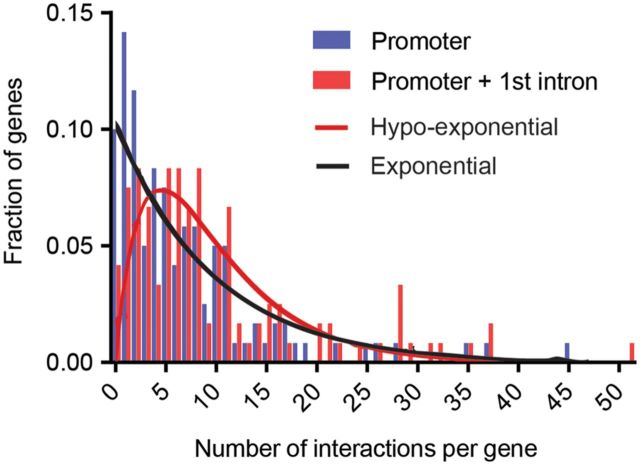
Network topology. Distribution of the number of TF interactions by gene determined by eY1H only considering promoter interactions or considering promoter and first intron interactions. The expected hypo-exponential and exponential for the gene in-degree considering both promoter and first intron interactions are shown.

Several studies in bacteria, yeast and *C. elegans* have shown that the number of TF interactions (in-degree) per promoter best follows an exponential distribution, with most promoters binding very few TFs and a few promoters binding a disproportionately large number of TFs (20,21,32,33). Given that TFs that bind the promoter and first intron are largely non-overlapping, we evaluated the gene in-degree distribution taking both DNA regions into account. When we considered promoter as well as first intron interactions obtained by eY1H, we found that the distribution of TF interactions per gene is less skewed than when only promoter interactions are taken into account (Figure 5). The distribution obtained when summing two or more variables randomly sampled from independent exponential distributions, such as the in-degree distributions for TF-promoter and TF-first intron interactions (Figures 3 and 5), has been shown to follow a hypo-exponential distribution (34). Consistent with this, results depicted in Figure 5 show that when promoter and first intron interactions are both considered, the gene in-degree follows a hypo-exponential rather than exponential distribution. In general, the greater the number of exponential distributions summed, the more symmetrical the overall distribution is. This suggests that including other regulatory regions may result in a more symmetrical distribution of the number of TFs that interact with each gene than previously considered. Future studies with additional regulatory regions in *C. elegans* as well as in other organisms will illuminate the generality of this observation.

### Gene families with long first introns

We found that long first introns (>250 bp) often occur in specific gene families. For instance, GPCR, the endoglin/CD105 protein family, homeodomain TFs and neurotransmitter-gated ion channels on average have long first introns (Table 1). Importantly, this is not a general feature for all introns in these families, as non-first introns are, in general, not longer than those of other genes (Table 1). Additionally, no family enrichment was detected within the group of genes with long second introns (data not shown).

**Figure 6.**
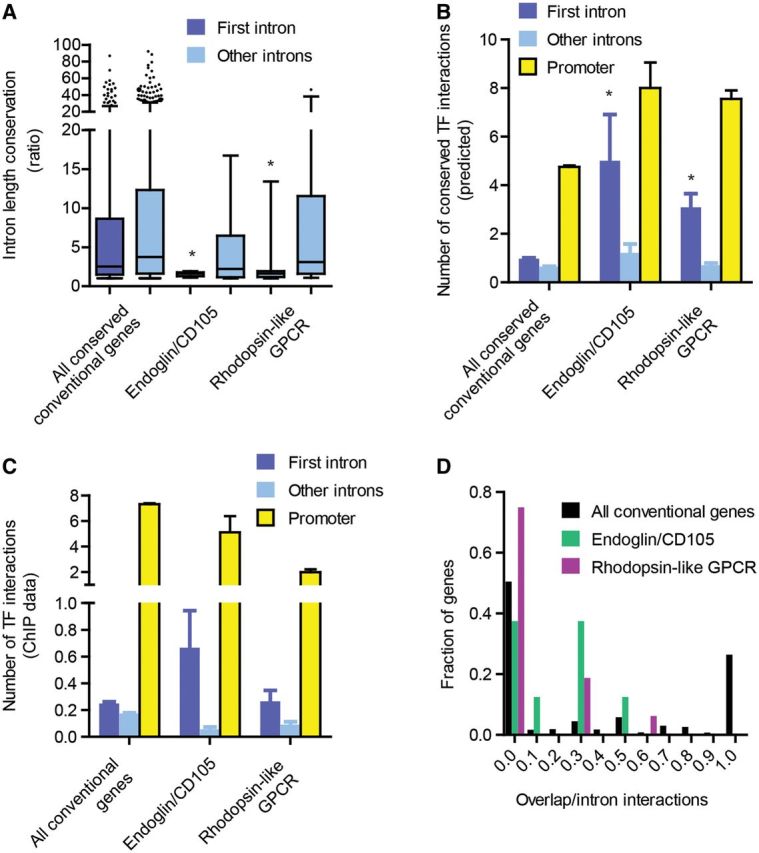
Role of first introns in TF binding for the endoglin/CD105 and GPCR, rhodopsin-like gene families. (**A**) Ratio between *C. elegans* and *C. briggsae* intron lengths (larger over smaller) determined between orthologous introns of orthologous genes for the endoglin/CD105 and rhodopsin-like GPCR gene families. (**B**) Average number (±SEM) of predicted TF interactions that are conserved between *C. elegans* and *C. briggsae* orthologous gene regions. (**C**) Average number (±SEM) of TFs that interact with each region obtained from ChIP assays. (**D**) Fraction of shared TF interactions determined by ChIP between first introns and promoters. *P < 0.05 versus first introns of all conserved conventional genes, Mann–Whitney *U* test.

To analyze the importance of first introns in TF binding for these gene families, we selected the two families with longest first introns for a more in-depth analysis: the endoglin/CD105 and the rhodopsin-like GPCR families. Both families exhibit a higher conservation in first intron length between *C. elegans* and *C. briggsae* than the rest of the conventional genes (Figure 6A). Additionally, for these families, first introns are more conserved in length than other introns. Further, first introns of these two families have a significantly higher number of conserved predicted TF interactions than first introns of all conventional genes (Figure 6B). This suggests that first introns of the endoglin/CD105 and the rhodopsin-like GPCR families are under more selective pressure than first introns of other genes and other introns for the same families.

The endoglin/CD105 family has a 3-fold higher number of first intron interactions determined by ChIP assays than first introns of other genes (Figure 6C). This increased binding is specific for the first intron as other introns show low TF binding. Conversely, the rhodopsin-like GPCR family has an average number of first intron interactions, comparable with that of other genes. Although this could be related to a relatively lower impact of first introns in gene regulation for the latter family, it is more likely related to a low number of TFs in the ChIP dataset binding to regulatory regions of rhodopsin-like GPCR genes (average promoter interactions of 2.0 versus 7.3 for all conventional genes)(Figure 6C). As observed for all conventional genes, there is little redundancy between first intron and promoter interactions for both gene families (Figure 6D). Together, these results suggest that first introns of the rhodopsin-like GPCR and endoglin/CD105 gene families may play an important role in gene regulation. In worms, most members of the gene families with long first introns are predominantly expressed in neurons and/or during development (www.wormbase.org). Interestingly, in mice, neuronal and developmental genes have been associated with the greatest enrichment of putatively functional noncoding sequences in first introns as defined by sequence constraint, suggesting a more complex regulation for this set of genes (35). Thus, long first introns harboring regulatory elements may be a conserved feature in a subset of neuronally expressed genes in metazoa.

## CONCLUSIONS

In this study, we investigated the role of *C. elegans* first introns in gene regulation by combining a systems-level gene-centered PDI network, computational predictions and previously published ChIP and microarray expression data. Our results suggest that first introns are under selective pressure and have more TF interactions than other introns, with more than half of *C. elegans* conventional gene first introns predicted to be involved in TF interactions.

We show that TF interactions with first introns are not redundant with interactions found in the promoter. A recent transgene expression study in *C. elegans* found that, when comparing the expression pattern using promoter::reporter constructs with the expression pattern obtained using tagged fosmid transgenes that preserve native *cis*-regulatory elements, introns and untranslated region sequences, only 62% of the expressing cells overlapped (45). The authors propose that these differences are potentially related to additional *cis*-regulatory elements in the fosmid transgene constructs and/or to posttranslational control. Consistent with this, we found that including first intron interactions in addition to promoter interactions helps predict gene co-expression based on PDI similarity.

Our study shows that first introns of *C. elegans* genes have a significant role in TF binding and can have an impact on gene regulation, in particular for those gene families that have long first introns. This should be taken into consideration when delineating gene regulatory networks.

## SUPPLEMENTARY DATA

Supplementary Data are available at NAR Online.

## FUNDING

National Institutes of Health (NIH) grants [DK068429, GM082971 to A.J.M.W.] and a postdoctoral fellowship from the Pew Latin American Fellows Program (to J.I.F.B. in part). Funding for open access charge: NIH.

*Conflict of interest statement*. None declared.

## Supplementary Material

Supplementary Data
