# Supplementary material for: Transcription factor binding to Caenorhabditis elegans first introns reveals lack of redundancy with gene promoters
Source: Nucleic Acids Res. 2013 Sep 24;42(1):153–62. doi: 10.1093/nar/gkt858 (PMC3874175; doi:10.1093/nar/gkt858)
Supplement: Supplementary Data [file supp_42_1_153__index.html]

Transcription factor binding to Caenorhabditis elegans first introns reveals lack of redundancy with gene promoters — Transcription factor binding to Caenorhabditis elegans first introns reveals lack of redundancy with gene promoters — Supplementary Data 

# Transcription factor binding to *Caenorhabditis elegans* first introns reveals lack of redundancy with gene promoters

## Supplementary Data

files

**Files in this Data Supplement:**

- Supplementary Data - xlsx file
- Supplementary Data - xlsx file
